# Predicting *in vivo* activity of combination therapies from *in vitro* drug pairs in diverse environments

**DOI:** 10.1016/j.xcrm.2022.100745

**Published:** 2022-09-20

**Authors:** Sarah Patterson, Adam Palmer

**Affiliations:** 1 Department of Pharmacology, University of North Carolina at Chapel Hill, Chapel Hill, NC, USA

## Abstract

New antibiotic combinations are needed to improve the treatment of tuberculosis. Larkins-Ford and colleagues share a framework that combines *in vitro* pairwise drug response data and machine learning to rationally prioritize combinations for clinical development.^1^

## Main text

Tuberculosis has been a global health emergency for the past 25 years, with more than 10 million new infections a year.^2^ The current standard treatment is a combination of four antibiotics that patients receive for up to 9 months. Despite this prolonged multi-drug therapy, antibiotic resistance develops in around 10% of cases (over a million people a year), so superior antibiotic combinations are sorely needed to increase the efficacy and shorten treatment time for patients.

The development of 30+ new antibiotics for tuberculosis is a mixed blessing: the space of possible multi-drug regimens is too vast to test in human studies, or even in mice.^3^ Therefore, strategies are needed to prioritize drug combinations for *in vivo* and clinical development.

**Figure 1:**
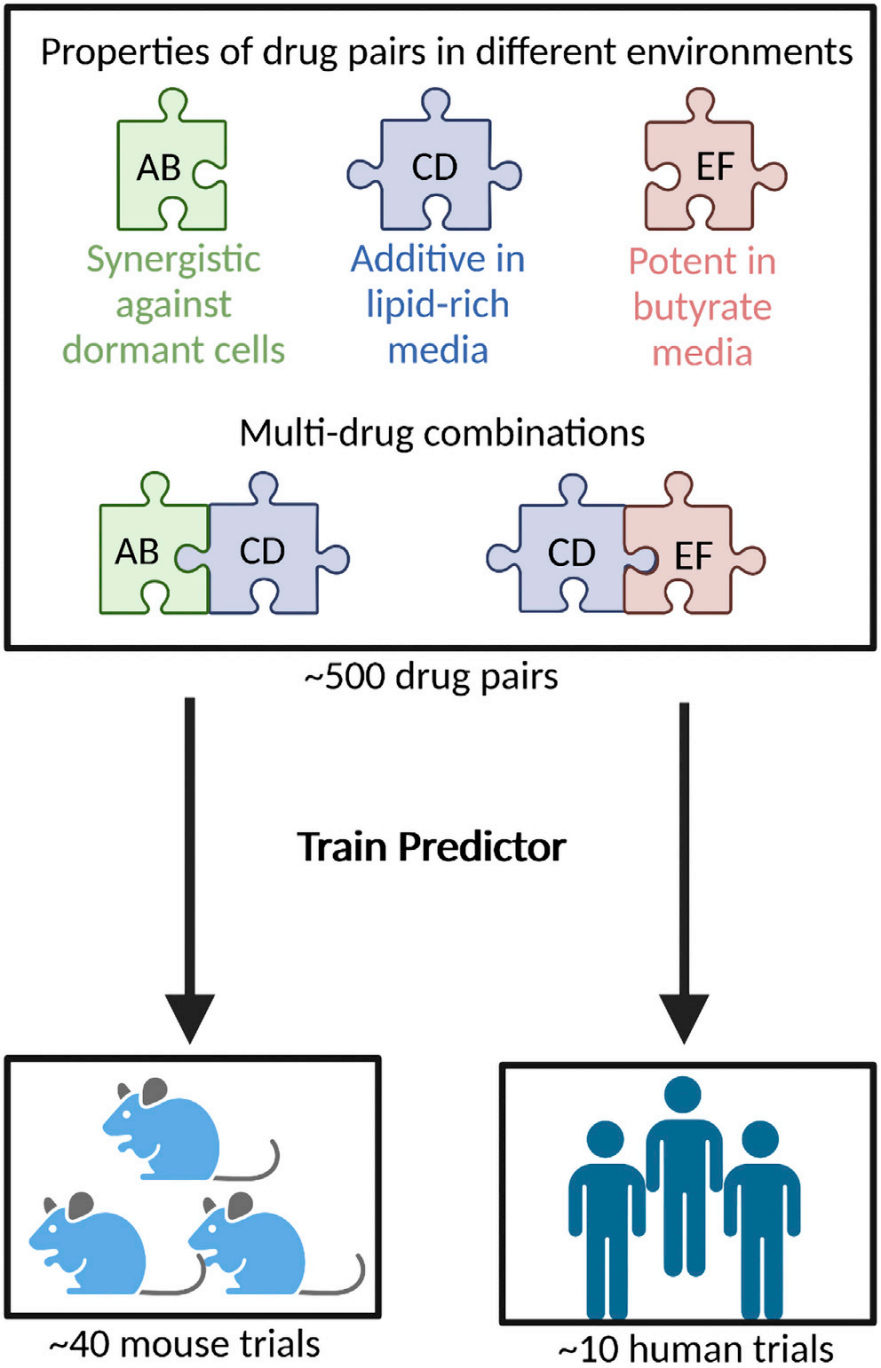
Combination therapy design strategy uses *in vitro* drug pair response with machine learning trained on mouse and human data Top: pairwise drug combinations (AB, CD, EF) have varying properties, such as potencies and drug interactions, across different growth conditions *in vitro*, which can represent diverse physiological environments. Promising combinations have drug pairs that are active across multiple growth states. Bottom: combination therapy data from mice and humans were used to train two separate models to predict whether candidate combinations were likely to be better than a standard of care regimen, based from the *in vitro* drug responses.

Larkins-Ford used pairwise drug response data in multiple *in vitro* growth environments, coupled with existing results of novel regimens tested in mice or humans, to train machine learning models to predict *in vivo* efficacy from *in vitro* properties of drug pairs (Figure 1).^1^ This publication is a continuation of a previous study where Larkins-Ford and colleagues tested antibiotic combinations across different growth environments.^4^ They discovered that while no drug pair was the best for all environments, effective regimens contain drugs whose collective activity spans diverse environments, particularly dormant growth conditions where tuberculosis is hardest to kill. Here, this new publication expands the *in vitro* response data to 12 antibiotics, testing 60 drug pairs across 7 different growth environments. Again, growth conditions that induce drug-refractory states (lipid-rich and non-replicating/dormant states) were among the best indicators of *in vivo* and clinical potency.

The authors trained their machine learning algorithm to predict the *in vivo* efficacy of combinations of 3–5 drugs, which had been previously tested in ∼40 mouse experiments and ∼10 phase 2 human trials. The algorithm used a common technique, random forest, to classify each candidate drug combination as being better or worse than the standard of care. Consistent with existing mouse and human trials, the results suggest that finding superior combinations is not searching for a “needle in a haystack”, but rather, many combinations of novel agents may be able to improve efficacy.

Given many candidate combinations, future pre-clinical development could prioritize the top performing “hits”, such as the top 10% of combinations. There is likely to be substantial redundancy among top candidates, for example, many regimens could contain the same drug pair that is synergistic against dormant tuberculosis. Therefore, the search space of possible combinations should be more manageable than raw numbers suggest. As a result, the system successfully reduces thousands of combination choices to tens—an amount that can be more practically evaluated.

Unsurprisingly, past trials have found that not all combinations with promise in mouse models prove effective in humans. To address these discrepancies, Larkins-Ford and colleagues trained separate random forest models for mouse and human data. Decision trees produced interpretable design principles, which for some environments emphasized potency, and in other environments, drug-drug interactions were important. Several principles were shared between the mouse and human trained models, particularly, which *in vitro* growth environments were best predictive of *in vivo* outcomes. However, there are substantial differences in predicted success in mice or humans. Ultimately, the ability to predict the results of mouse experiments is chiefly a proof of concept for the most important task of predicting efficacy in humans. On the basis of these results, we are optimistic that this task should be increasingly possible as training data from human trials grows.

This study further advances a conception of high-order combination regimens as a set of drug pairs with unique characteristics. Studies in a variety of bacteria and cancer have consistently shown that dose responses of high-order drug combinations can be predicted from their pairwise drug interactions, showing again that both potency and drug interactions are important when designing antibiotic combinations.^5^, ^6^, ^7^ In the present article, promising drug combinations commonly included potent drug pairs, but one pair alone was not responsible for predicting the overall effect of a combination. Each pair within a regimen seemed to add a unique benefit to a combination like building blocks, an idea represented in Figure 1. Different pairs were potent in different growth environments, and strong combinations diversified their pairwise potencies across multiple growth environments to maximize bactericidal activity. Speculatively, it may be ideal to have at least two highly active agents for every physiologically relevant environment, in order to thwart the evolution of drug resistance in all circumstances.

The studies by Larkins-Ford and colleagues indicate that training machine learning on matched *in vitro* and *in vivo* experiments can help to bridge this difficult step in the development of new drug combinations. The framework could in principle be applied to different diseases where drug combinations are used, including cancer and other pathogens.^8^^,^^9^ The primary caveat of this approach is that existing combination response data are required to train an accurate model, and in many diseases a limited number of drug combinations have been tested in human trials. Overall, this study explores a new system pairing *in vitro* drug responses with *in vivo*-trained machine learning to select drug regimens with the best likelihood of clinical success.

## References

[1] Larkins-Ford J, Degefu Y.N. , Van N. , Sokolov A., Aldridge B.B. Design principles to assemble drug combinations for effective tuberculosis therapy using interpretable pairwise drug response measurements. Cell Rep. Med. *3,* 100737. 10.1016/j.xcrm.2022.100737 PMC9512659 36084643

[2] Furin J., Cox H., Pai M. (2019). Tuberculosis. The Lancet..

[3] Peloquin C.A., Davies G.R. (2021). The treatment of tuberculosis. Clin. Pharmacol. Ther..

[4] Larkins-Ford J., Greenstein T., Van N., Degefu Y.N., Olson M.C., Sokolov A., Aldridge B.B. (2021). Systematic measurement of combination-drug landscapes to predict in vivo treatment outcomes for tuberculosis. Cell Syst..

[5] Wood K., Nishida S., Sontag E.D., Cluzel P. (2012). Mechanism-independent method for predicting response to multidrug combinations in bacteria. Proc. Natl. Acad. Sci. USA.

[6] Katzir I., Cokol M., Aldridge B.B., Alon U. (2019). Prediction of ultra-high-order antibiotic combinations based on pairwise interactions. PLoS Comput. Biol..

[7] Zimmer A., Katzir I., Dekel E., Mayo A.E., Alon U. (2016). Prediction of multidimensional drug dose responses based on measurements of drug pairs. Proc. Natl. Acad. Sci. USA.

[8] Boshuizen J., Peeper D.S. (2020). Rational cancer treatment combinations: an urgent clinical need. Mol. Cell.

[9] Shyr Z.A., Cheng Y.S., Lo D.C., Zheng W. (2021). Drug combination therapy for emerging viral diseases. Drug Discov. Today.

